# Mortality outcomes of low-dose computed tomography screening for lung cancer in urban China: a decision analysis and implications for practice

**DOI:** 10.1186/s40880-017-0221-8

**Published:** 2017-07-14

**Authors:** Zixing Wang, Wei Han, Weiwei Zhang, Fang Xue, Yuyan Wang, Yaoda Hu, Lei Wang, Chunwu Zhou, Yao Huang, Shijun Zhao, Wei Song, Xin Sui, Ruihong Shi, Jingmei Jiang

**Affiliations:** 1 Institute of Basic Medical Sciences, Chinese Academy of Medical Sciences, Beijing, 100005 P. R. China; 2 Cancer Hospital, Chinese Academy of Medical Sciences, Beijing, 100021 P. R. China; 3 Peking Union Medical College Hospital, Chinese Academy of Medical Sciences, Beijing, 100730 P. R. China; 4 National Institutes for Food and Drug Control, State Food and Drug Administration, Beijing, 100050 P. R. China

**Keywords:** Lung cancer, Low-dose CT, Screening, Mortality outcome, Decision analysis

## Abstract

**Background:**

Mortality outcomes in trials of low-dose computed tomography (CT) screening for lung cancer are inconsistent. This study aimed to evaluate whether CT screening in urban areas of China could reduce lung cancer mortality and to investigate the factors that associate with the screening effect.

**Methods:**

A decision tree model with three scenarios (low-dose CT screening, chest X-ray screening, and no screening) was developed to compare screening results in a simulated Chinese urban cohort (100,000 smokers aged 45–80 years). Data of participant characteristics were obtained from national registries and epidemiological surveys for estimating lung cancer prevalence. The selection of other tree variables such as sensitivities and specificities of low-dose CT and chest X-ray screening were based on literature research. Differences in lung cancer mortality (primary outcome), false diagnoses, and deaths due to false diagnosis were calculated. Sensitivity analyses were performed to identify the factors that associate with the screening results and to ascertain worst and optimal screening effects considering possible ranges of the variables.

**Results:**

Among the 100,000 subjects, there were 448, 541, and 591 lung cancer deaths in the low-dose CT, chest X-ray, and no screening scenarios, respectively (17.2% reduction in low-dose CT screening over chest X-ray screening and 24.2% over no screening). The costs of the two screening scenarios were 9387 and 2497 false diagnoses and 7 and 2 deaths due to false diagnosis among the 100,000 persons, respectively. The factors that most influenced death reduction with low-dose CT screening over no screening were lung cancer prevalence in the screened cohort, low-dose CT sensitivity, and proportion of early-stage cancers among low-dose CT detected lung cancers. Considering all possibilities, reduction in deaths (relative numbers) with low-dose CT screening in the worst and optimal cases were 16 (5.4%) and 288 (40.2%) over no screening, respectively.

**Conclusions:**

In terms of mortality outcomes, our findings favor conducting low-dose CT screening in urban China. However, approaches to reducing false diagnoses and optimizing important screening conditions such as enrollment criteria for screening are highly needed.

## Background

Lung cancer is the most common malignant tumor and the dominant cause of cancer-related deaths in China [1]. The 5-year overall survival rate of patients with lung cancer remains approximately 15%–18% even in developed countries [2], whereas for patients who undergo surgical resection of stage I cancer this rate is well above 70% [3], highlighting the urgent need for early detection and treatment. First introduced in the 1990s, low-dose computed tomography (CT) has become the most promising approach for lung cancer screening [4]. Increasing number of screening programs in North America [5], Japan [6], and Europe [7–14] have greatly augmented the volume of the evidence base concerning low-dose CT screening practice. However, in China, a country with 36% of all lung cancers worldwide [15], mortality outcome, which is the most important measure for assessing screening effects, has not yet been evaluated despite numerous preliminary studies on diagnostic accuracy [16].

The United States National Lung Screening Trial (NLST) has reported an encouraging 20% reduction in lung cancer mortality with low-dose CT screening over chest X-ray screening [5]. However, uncertainties remain regarding the mortality outcomes of screening programs because of controversial results from other countries [7–14]. Two randomized controlled trials (RCTs) from Italy [7, 8] and one from Denmark [9] reported equal or slightly increased mortalities (not statistically significant) for low-dose CT screening compared with no screening. Five ongoing RCTs (from Germany [10], Italy [11], France [12], the Netherlands, Belgium and Hungary [13], and the United Kingdom [14]) have not yet released their mortality outcomes. Outcomes of screening programs may vary between settings because of diversity in participants’ characteristics and healthcare service conditions [17]. Therefore, there is a need to investigate whether early detection by low-dose CT screening could reduce lung cancer mortality in China and to investigate the variations in screening effects before introducing such screening countrywide. This study aimed to analyze differences in lung cancer mortality between three scenarios using available data from China and to identify factors that most strongly influence the outcomes in low-dose CT screening.

## Methods

### Study population

In this study, we simulated a cohort of 100,000 urban residents and offered them one-off lung cancer screening (baseline screening, i.e., prevalence screening). The age and sex structures of this cohort were based on data from the China Population & Employment Statistics Yearbook 2014 [18]. According to data on lung cancer incidence reported by the National Cancer Registry 2012 [19], age criterion for this cohort was set at 45–80 years. As there is currently no high-quality evidence-based recommendation on inclusion criteria for lung cancer screening in China [20], we adopted the relatively relaxed criterion of “smokers” as a requirement of the 100,000 participants, regardless of daily smoking dose, smoking years, and cumulative pack-years (this choice of criterion was made also because it was used in national surveys on smoking in China [21]). No other restrictions were imposed such as family history of lung cancer or individual history of pulmonary disease. However, we did exclude lung cancer screening for special categories, such as patients with tuberculosis or human immunodeficiency virus infection and individuals with work-place exposure to asbestos, coal dust, nuclear radiation, organic solvents, and fuels.

### Decision tree model

**Fig. 1:**
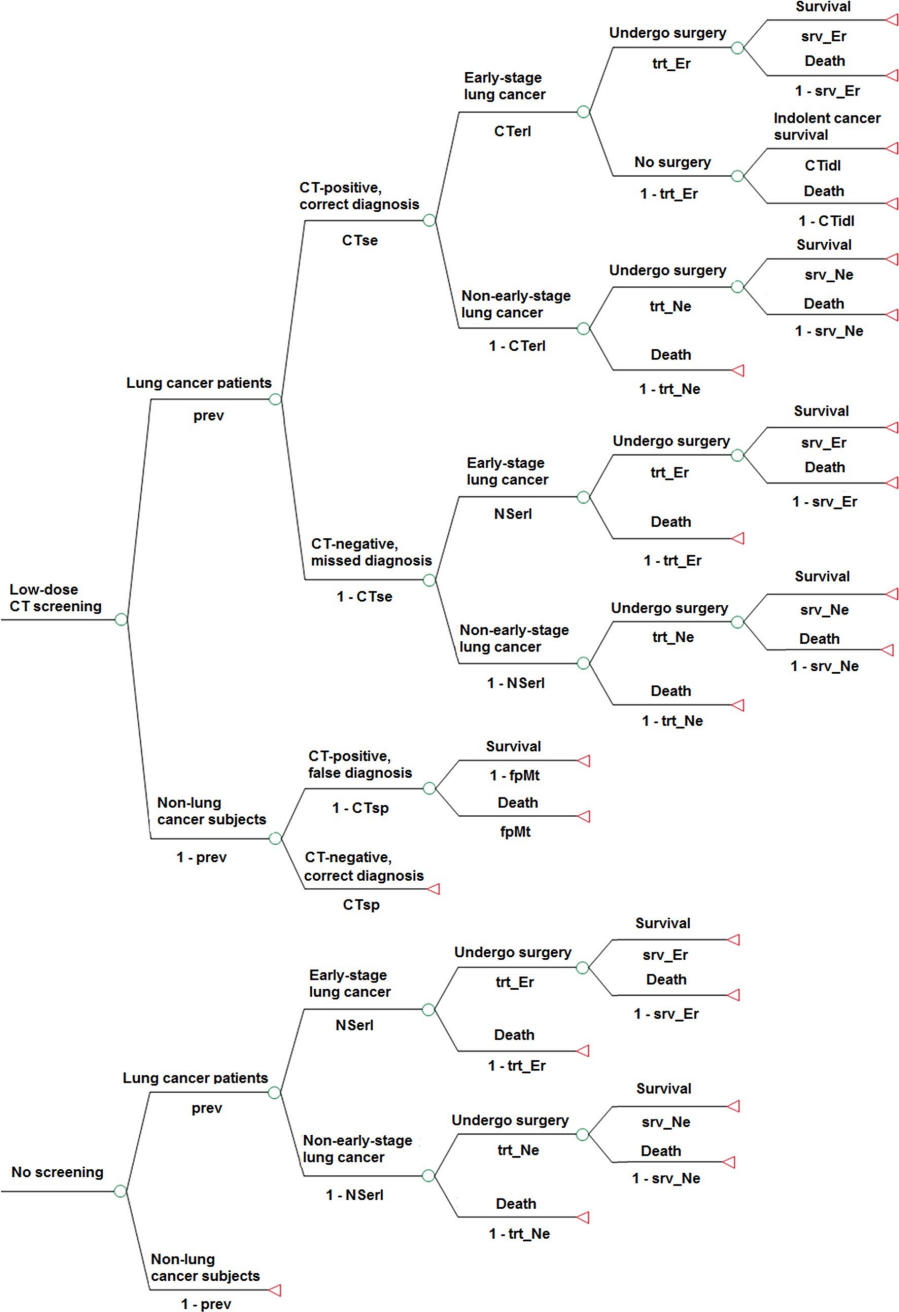
Decision tree model for the analysis of lung cancer mortality, false positive diagnosis, and death due to false diagnosis with low-dose computed tomography (CT) screening and no screening. Chest X-ray screening is similar to low-dose CT screening and is not shown. *prev* lung cancer prevalence in the screened cohort, *CTse* sensitivity of low-dose CT, *CTsp* specificity of low-dose CT, *CTerl* proportion of early-stage cancers among lung cancers detected with low-dose CT, *NSerl* proportion of early-stage cancers among lung cancers detected with no screening, *fpMt* death possibility due to false diagnosis and invasive treatment, *trt_Er* acceptance rate of surgery for individuals with early-stage lung cancers, *trt_Ne* acceptance rate of surgery for individuals with non-early-stage lung cancers, *srv_Er* survival possibility of individuals with resected early-stage lung cancer, *CTidl* proportion of indolent cancers among lung cancers detected with low-dose CT, *srv_Ne* survival possibility of individuals with resected non-early-stage lung cancer

Decision analysis is widely used to make choices between different paths in Chaotics and uncertain conditions [22], especially in healthcare studies where medical outcomes such as mortality depend on a great number of potentially influencing factors [23]. To identify the best approach to detecting lung cancer so as to improve outcomes and to test the degree of uncertainty, we considered and compared the following three possible paths for lung cancer detection and treatment (Fig. 1).

#### Low-dose CT screening scenario

Individuals with findings suggestive of lung cancer on low-dose CT images were asked to undergo further investigation, such as bronchoscopy and percutaneous biopsy, to determine the diagnosis and then either accept or refuse surgical treatment. Additionally, we set up branches in the path corresponding with “missed diagnosis” caused by false negative imaging results (merging into the no screening path described below), “false diagnosis” caused by false positive imaging results, and the “over diagnosis” issue (individuals with indolent cancer who live for a long time though untreated) recently raised by researchers [24].

#### Chest X-ray screening scenario

The overall path was similar to that in the low-dose CT screening scenario, with the only difference being that the variables were adjusted to chest X-ray screening.

#### No screening scenario

Individuals with lung cancer detected and further diagnosed according to symptoms or medical examinations for other diseases took the usual health care treatment path.

All the three scenarios were stratified by early (stage I for non-small cell lung cancers and limited stage for small cell lung cancers) and non-early stage cancers, considering the impact of disease stage on the acceptance rate of surgery and survival possibility after surgery.

Additionally, the following assumptions were made for the decision tree. First, surgery is a determinant for lung cancer mortality (except for indolent cancers), and although non-surgical treatments could prolong patient survival, their small impact on the final outcomes was not taken into account [25]. Second, a 5-year time span was adopted. As the screened cohort had a high competing risk from other diseases, deaths beyond the 5-year range were not considered in the outcome evaluation.

### Parameter setting

Table 1 lists all the variables and the estimated values of parameters used in the decision tree. Specifically, the selection of screening parameters of low-dose CT and chest X-ray and the clinical parameters of diagnosis and treatment path were based on a systematic search of literatures (mainly from studies in China, together with a few studies in other countries, all extracted from MEDLINE, EMBASE, CNKI, and ChinaInfo databases and published between January 1990 and February 2016). Quality-weighted meta-analyses were performed to obtain the average estimate of parameter for each tree variable for base-case analysis (analysis based on the most likely estimates of parameters) and a reliable range for sensitivity analysis.

**Table 1:** Parameter settings in the decision tree for lung cancer screening in urban China

Variable	Definition	Base-case analysis (%)	Sensitivity analysis (%)		Reference source
			Lower limit	Upper limit	
prev	Lung cancer prevalence in the screened cohort	0.7666	0.3833	0.8943	Calculated from [18, 22, 26, 32, 53]
CTse	Sensitivity of low-dose CT	87.7	71.8	100.0	Meta of [21, 54–66]
CTsp	Specificity of low-dose CT	90.6	86.3	91.1	Meta of [55, 57–60, 63, 66–69]
XRse	Sensitivity of chest X-ray	65.1	61.4	69.4	Meta of [21, 55–57, 60, 62–65, 70]
XRsp	Specificity of chest X-ray	97.5	89.5	98.4	Meta of [55, 57, 60, 63, 69, 71, 72]
CTerl	Proportion of early-stage cancer^a^ among lung cancers detected with low-dose CT	70.1	63.9	76.0	[73]
XRerl	Proportion of early-stage cancer^a^ among lung cancers detected with chest X-ray	46.6	38.1	55.0	[73, 74]
NSerl	Proportion of early-stage cancers^a^ among lung cancers detected with no screening	27.9	23.2	32.7	Meta of [24, 55, 76–79 52 ^d^, 75 ^e^, ]
CTidl	Proportion of indolent cancers^b^ among lung cancers detected with low-dose CT	25.0	15.0	35.0	[20]
XRidl	Proportion of indolent cancers^b^ among lung cancers detected with chest X-ray	20.0	15.0	25.0	[20]
trt_Er	Acceptance rate of surgery for early-stage lung cancers^a^	72.5	68.2	76.0	Meta of [80–82]
trt_Ne	Acceptance rate of surgery for non-early-stage lung cancers^a^	28.6	28.2	30.6	Meta of [80–82]
srv_Er	Survival possibility of individuals with resected early-stage lung cancer^c^	72.7	62.3	75.7	Meta of [3, 35, 83]
srv_Ne	Survival possibility of individuals with resected non-early-stage lung cancer^c^	39.7	30.2	42.4	Meta of [3, 35, 83]
fpMt	Death possibility due to false diagnosis and invasive treatment	0.07	0.00	0.13	[84]

*CT* computed tomography

^a^Stage I for non-small cell lung cancers and limited stage for small cell lung cancers were defined as early-stage cancers, and others as non-early-stage cancers

^b^Lung cancers such as bronchioloalveolar carcinoma that did not affect survival for a long time if left untreated were defined as indolent cancers

^c^Based on 5-year estimation

^d^Seven studies that were conducted among the Chinese population with complete stage information [85–91] in this meta-analysis were included and reanalyzed in this study

^e^Eight studies with complete stage information [92–99] in this meta-analysis were included and reanalyzed in this study

Lung cancer prevalence of the screened cohort was calculated as follows.

First, age- and sex-specific lung cancer incidences in the general Chinese urban population ($I_{\text{G}}$) were extracted from the China Cancer Registry 2012 (urban data) at age intervals of 5 years [19]. Next, the incidence of the screened cohort ($I_{\text{S}}$) for each age and sex group was calculated using the formula below:

$$I_{\text{S}} = \frac{{{\text{OR}} \times I_{\text{G}} }}{{1 + ({\text{OR}} - 1) \times R}}$$

where *R* is the age- and sex-specific rate of smoking reported in the Global Adult Tobacco Survey (GATS) China 2010 Country Report [26] and OR (2.85 for men and 2.33 for women) is the odds ratio according to meta-analysis results based on five case–control studies in China that used newly incident lung cancers to estimate the degree of association between smoking and lung cancer between 2001 and 2014 [27–31].

Further, because lung cancer prevalence is relatively stable in China, the following formula was used to calculate age- and sex-specific lung cancer prevalence in the screened cohort ($P_{\text{S}}$):

$$P_{\text{S}} = I_{\text{S}} \times t$$

where *t* = 3.0 years (sensitivity range 1.5–3.5 years) is the average course of lung cancer [32].

Finally, the overall prevalence of lung cancer in the screened cohort aged 45–80 years was calculated by standardizing the age- and sex-specific prevalence using the actual demographical structure reported in the China Population & Employment Statistics Yearbook 2014 [18] and the smoking rates in the GATS China 2010 Country Report [26].

Quality-adjusted life year (QALY) [33] was analyzed for the three scenarios over a 5-year time span. Quality utilities that were incorporated into the model were 0.76 for individuals without lung cancer [34], 0.62 for the time from detection of lung cancer to surgical treatment [35], 0.67 for early- and 0.55 for non-early-stage cancer patients after surgical treatment [35], and 0.56 for those who refused surgical treatment after detection, regardless of cancer stage and presence of other treatments [35]. We used an average delay of 0.5 years to lung cancer detection in the no screening scenario compared with the two screening scenarios, and incorporated an interval of 0.25 years for all the individuals with lung cancer from detection to surgical treatment (by expert consultation). The mean survival within the 5-year time span was estimated as 4.31 and 2.99 years for individuals with resected early- and non-early-stage cancers, respectively [3], and as 2.78 and 0.71 years for individuals with non-resected early- and non-early-stage cancers, respectively [36].

Additionally, to explore the influences of criteria of age, smoking, and sex on the prevalence of lung cancer in the screened cohort (and therefore on screening outcomes), with the same procedures described above, we further calculated different ${\text{P}}_{S}$ for 12 plausible sub-intervals within the 45–80-year age range to determine the best lower and upper age limits in terms of mortality, and ${\text{P}}_{S}$ for men and women separately, each with 11 different proportions of smoking among the enrolled individuals if screening was not restricted to smokers.

### Statistical analysis

Lung cancer death was the primary outcome measure in this study. Using base-case analysis, we calculated the absolute and relative reductions in lung cancer deaths with low-dose CT screening over chest X-ray screening and no screening. False diagnosis, death due to false diagnosis, and QALY as secondary outcome measures were simultaneously calculated and compared. Univariate sensitivity analysis (by separately letting each variable in the model fluctuate within its lower and upper ranges for sensitivity analysis while keeping the others at their base-case analysis values) was performed to investigate the influences of screening conditions on outcomes in different scenarios, and a tornado diagram was plotted to vividly display the magnitude of the influence of each variable (relative importance of the variables was ranked by percentages of variations in the outcomes due to their own influence over the variation summary due to influence of all variables). Optimal and worst cases of reductions in lung cancer deaths with low-dose CT screening over other scenarios were determined by considering combinations of all possible ranges of the variables. All the analyses were performed with Treeage Pro 2011 software (TreeAge Software, Inc, Williamstown, MA, USA).

## Results

### Base-case analysis

Age and sex distribution of the simulated cohort in the base-case analysis is displayed in Table 2. Among these 100,000 urban smokers (94,012 men and 5988 women) aged 45–80 years, 721 men and 46 women had lung cancers. As to the outcomes in the three scenarios for the same cohort, the number of lung cancer deaths in the low-dose CT screening scenario was 448, a reduction of 143 (24.2%) over the no screening scenario (591 lung cancer deaths); meanwhile, low-dose CT screening resulted in 9387 false diagnoses and 7 deaths due to false diagnosis. In the chest X-ray screening scenario, there were 541 lung cancer deaths, a reduction of 50 (8.5%) over the no screening scenario, and chest X-ray screening resulted in 2497 false diagnoses and 2 deaths due to false diagnosis. There were 93 fewer lung cancer deaths (17.2%) in the low-dose CT screening scenario than in the chest X-ray screening scenario; however, false diagnoses and deaths due to false diagnosis were 3.76 and 3.50 times higher, respectively, in the former scenario. Additionally, QALYs were 378,427 years in low-dose CT screening, 378,280 years in chest X-ray screening, and 378,177 years in no screening scenarios, that is, low-dose CT screening resulted in slightly more QALYs: 147 years over chest X-ray screening and 250 years over no screening.

**Table 2:** Age and sex distribution of the simulated smoking cohort for base-case analysis

Age group (years)	General population structure (%)^a^		General population smoking rate (%)^b^		No. of smokers for base-case analysis (total = 100,000)		No. of lung cancer patients in 100,000 smokers for base-case analysis	
	Men	Women	Men	Women	Men	Women	Men	Women
45–49	13.4	12.6	69.5	2.9	29,489	1157	64	3
50–54	10.1	9.4	63.1	4.1	20,307	1224	88	4
55–59	9.3	9.3	61.9	3.0	18,356	884	123	5
60–64	6.8	7.1	54.9	2.5	11,793	566	126	4
65–69	4.4	4.7	45.7	8.1	6403	1200	109	13
70–74	3.2	3.5	42.3^c^	4.3^c^	4259	476	103	8
75–80	2.9	3.4	36.7^c^	4.5^c^	3405	481	108	9
Summary	50.1	49.9	59.1	3.8	94,012	5988	721	46

^a^Obtained from Sampling Survey Data of the National Population Change in 2013 in China Population & Employment Statistics Yearbook [18]

^b^Obtained from Global Adult Tobacco Survey (GATS) China 2010 Country Report [26]

^c^Predicted using linear regression

### Univariate sensitivity analysis

**Fig. 2:**
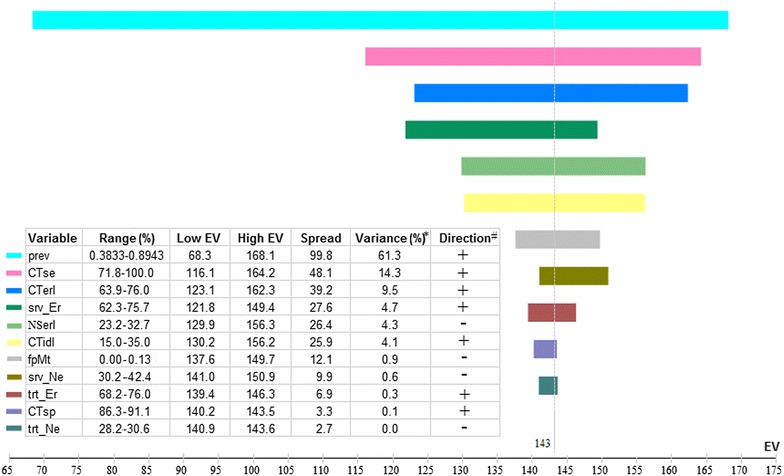
Tornado histogram of lung cancer deaths in the low-dose CT screening scenario over the no screening scenario. *EV* effect value, *horizontal line* represents the relative difference in lung cancer deaths with low-dose CT screening over no screening (the higher the EV, the higher the screening benefits), and *vertical dash line* indicates the location of estimated average EV, *prev* lung cancer prevalence in the screened cohort, *CTse* sensitivity of low-dose CT, *CTerl* proportion of early-stage cancers among lung cancers detected with low-dose CT, *srv_Er* survival possibility of patients with resected early-stage lung cancers, *NSerl* proportion of early-stage cancers among lung cancers detected with no screening, *CTidl* proportion of indolent cancers among lung cancers detected with low-dose CT, *fpMt* death possibility due to false diagnosis and invasive treatment, *srv_Ne* survival possibility of individuals with resected non-early-stage lung cancer, *trt_Er* acceptance rate of surgery for early-stage lung cancers, *CTsp* specificity of low-dose CT, *trt_Ne* acceptance rate of surgery for non-early-stage lung cancers. *Asterisk* the proportion of difference squared between low and high EV for each variable of the sum of differences squared for all variables. The *plus sign* denotes that the higher the variable value, the greater the reduction in lung cancer deaths with low-dose CT screening over no screening, and the *minus sign* denotes that the higher the variable value, the smaller the reduction in lung cancer deaths with low-dose CT screening over no screening

Lung cancer prevalence in the screened cohort was the factor that most strongly influenced the death reduction with low-dose CT screening over no screening (Fig. 2). The variation in prevalence (from 383.3 to 894.3 per 100,000) led to a great variation in the absolute reduction in lung cancer deaths (from 68 to 168), being responsible for 61.3% of the all-variable influence. Other six factors besides the lung cancer prevalence contributed positively to the death reduction benefit in the following order: low-dose CT sensitivity (14.3% of the all-variable influence), proportion of early-stage cancers among lung cancers detected with low-dose CT (9.5%), survival possibility of individuals with resected early-stage lung cancer (4.7%), proportion of indolent cancers among lung cancers detected with low-dose CT (4.1%), acceptance rate of surgery for early-stage lung cancers (0.3%), and specificity of low-dose CT (0.1%). These six factors together contributed 33.0% to the variation in reduction in lung cancer deaths. Four factors were negatively related with reduction in lung cancer deaths with low-dose CT screening over no screening, namely proportion of early-stage cancers among lung cancers detected with no screening (4.3%), possibility of death due to false diagnosis and invasive treatment (0.9%), survival possibility of patients with resected non-early-stage lung cancer (0.6%), and acceptance rate of surgery for non-early-stage lung cancers (0.01%); however, their cumulative influence was subtle (5.8%).

For the secondary outcome measures (not shown in figures), the factor that most influenced the number of false diagnosis was specificity of low-dose CT (negatively contributing 99.9% to variation in increased false diagnoses); lung cancer prevalence had a tiny negative influence (0.01%). Three factors were related to number of deaths due to false diagnosis with low-dose CT screening, namely, death possibility due to false diagnosis (93.0%, positive), low-dose CT specificity (7.0%, negative), and lung cancer prevalence (0.01%, negative). The factor that most influenced DALY was lung cancer prevalence (58.7%), followed by low-dose CT sensitivity (13.5%), low-dose CT specificity (11.0%), and proportion of early-stage cancers among lung cancers detected with low-dose CT (10.0%; all positive). Notably, a threshold of QALY was detected in lung cancer prevalence (436 per 100,000); the gain of QALY in low-dose CT screening over no screening would diminish if screening was performed among populations with a low prevalence of lung cancer.

### Worst and optimal case analysis

Considering the possible ranges of all influential factors, reductions in lung cancer deaths with low-dose CT screening over no screening were 281 versus 297 (16 absolute reduction, 5.4% relative reduction) in the worst case and 428 versus 716 (288, 40.2% reduction) in the optimal case, and those over chest X-ray screening were 281 versus 270 (−11, −4.1% reduction) in the worst case and 428 versus 669 (241, 36.0% reduction) in the optimal case.

### Influence of age criteria

The influences of upper and lower age limits on prevalence of lung cancer in the screened cohort and on differences in numbers of lung cancer deaths between the three scenarios are shown in Table 3. Relative to no screening, more than 200 fewer lung cancer deaths (24.5% relative reduction) could be achieved by setting the age criteria for low-dose CT screening at 55–75, 55–80, 60–70, 60–75, or 60–80 years. Similarly, these intervals for age criteria could lead to more than 130 fewer deaths (17.5% relative reduction) with low-dose CT screening over chest X-ray screening. However, some of these age intervals covered relatively small portions of the total lung cancer patients among the whole population of 45–80 years old, e.g., only 32.9% and 47.3% of patients with lung cancer would be covered if age criteria of 60–70 and 60–75 years were adopted, respectively. The age interval of 50–75 years currently recommended by the Chinese expert consensus for lung cancer screening [20] covered 76.0% of all lung cancer patients aged 45–80 years, but the reduction in lung cancer deaths with low-dose CT screening was relatively low, 109 (17.4%) and 164 (24.3%), respectively, as compared with chest X-ray screening and no screening.

**Table 3:** Prevalence of lung cancer among 100,000 smokers and predicted lung cancer deaths in low-dose CT, chest X-ray, and no screening scenarios with different age inclusion criteria

Age interval (years)	Lung cancer prevalence (per 100,000)	Target coverage (%)^a^	Lung cancer deaths (cases)			Death reduction [cases (%)]	
			Low-dose CT	Chest X-ray	No screening	Low-dose CT over chest X-ray	Low-dose CT over no screening
45–70	589.2	70.2	346	417	454	71 (17.0)	108 (23.8)
45–75	675.4	84.7	395	477	521	82 (17.2)	126 (24.2)
45–80	766.6	100.0	448	541	591	93 (17.2)	143 (24.2)
50–70	777.0	61.6	454	549	599	95 (17.3)	145 (24.2)
50–75	890.0	76.0	519	628	686	109 (17.4)	167 (24.3)
50–80	1009.4	91.3	588	712	778	124 (17.4)	190 (24.4)
55–70	967.8	49.5	564	683	746	119 (17.4)	182 (24.4)
55–75	1115.6	63.9	649	787	860	138 (17.5)	211 (24.5)
55–80	1270.4	79.3	738	896	980	158 (17.6)	242 (24.7)
60–70	1263.0	32.9	734	891	974	157 (17.6)	240 (24.6)
60–75	1469.2	47.3	852	1036	1133	184 (17.8)	281 (24.8)
60–80	1680.2	62.7	974	1185	1296	211 (17.8)	322 (24.8)

^a^Proportion of lung cancer patients within the listed age interval among all lung cancer patients aged 45–80 years in urban China

### Influence of smoking and sex criteria

**Fig. 3:**
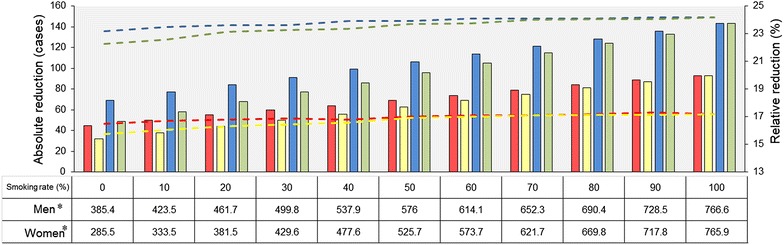
Reductions in lung cancer deaths with low-dose CT screening over chest X-ray screening and no screening among men and women of 45–80 years old with different proportions of smokers. *Bars* represent absolute reduction in deaths; *dotted lines* represent relative reduction in deaths. *Red* low-dose CT over chest X-ray screening for men, *yellow* low-dose CT over chest X-ray screening for women, *blue* low-dose CT over no screening for men, *green* low-dose CT over no screening for women. *Asterisk* lung cancer prevalence among the screened population, per 100,000

Figure 3 shows that when not restricted to smokers, the reduction in lung cancer deaths with low-dose CT screening over chest X-ray screening and no screening increased in parallel with the proportion of smokers in the screened cohort, both among men and women. For example, compared with no screening, the relative reduction in lung cancer deaths among men undergoing low-dose CT screening increased from 23.2% for non-smokers to 24.2% for smokers, and the absolute screening benefit doubled from 69 to 143 fewer deaths. Similarly, the absolute benefit gained by low-dose CT screening also doubled compared with chest X-ray screening (from 45 to 93 fewer deaths) for men. Notably, among women the relative reduction in lung cancer deaths with low-dose CT screening was as great as that for men when screening 100% smokers (24.2% over no screening and 17.2% over chest X-ray screening), and the absolute reduction in lung cancer deaths with low-dose CT screening almost tripled both compared with no screening (from 49 in non-smokers to 143 in smokers) and chest X-ray screening (from 32 to 93).

## Discussion

This study evaluated the outcomes of lung cancer screening in China, including the influences of several factors on screening effects. According to the base-case analysis of 100,000 urban smokers aged 45–80 years, low-dose CT screening decreased lung cancer deaths by 24.2% over no screening and by 17.2% over chest X-ray screening. There were fewer deaths even in the worst case analysis (lung cancer deaths of 245 vs 269 for low-dose CT screening and no screening, respectively; 8.9% relative reduction), and the reduction was as high as 43.6% (lung cancer deaths of 364 vs 645 in low-dose CT screening and no screening, respectively) in an optimal situation. Thus, in terms of mortality outcome, our findings indicate that screening old smokers in urban China with low-dose CT would reduce lung cancer deaths. However, the effect of low-dose CT screening on lung cancer death reduction was inferior to that of chest X-ray screening in the worst situation (lung cancer deaths of 281 vs 270 for low-dose CT and chest X-ray screenings, respectively; −4.1% relative reduction). On average, low-dose CT screening resulted in more than three-fold false diagnoses and even deaths due to false diagnosis and over-treatment. Additionally, the gain in QALYs with low-dose CT screening (250 years over no screening and 147 years over chest X-ray screening) was not as pronounced as that for mortality. Thus, better screening strategies are required to optimize the outcomes of low-dose CT screening.

Being a country with a large lung cancer burden (with more than 3,500,000 new cases and more than 2,000,000 fatal cases each year [37]), China has a considerable interest in the detection of early-stage lung cancer and prevention of lung cancer deaths via screening [16, 17]. Several studies have published preliminary results for the comparative ability of low-dose CT screening over chest X-ray screening to detect lung cancer and for other screening variables such as screening-detected cancer stage, pathological, and individual characteristics [16, 38]. In 2010, a demonstration program was initiated at three centers to test the feasibility of conducting population-based screening [39]. Two years later, the government incorporated urban lung cancer screening into a key national public health program [16]. Since 2013, approximately 8000 Beijing citizens have been screened in another program for 3 years using low-dose CT to optimize the screening protocol [16]. Most studies have reported rates of detection, early diagnosis, and early treatment as the main outcomes; however, none have yet reported the endpoint of lung cancer mortality, both because it takes so long to obtain this important but time-consuming endpoint of screening and because a great many participants are needed to identify mortality differences at a low cancer prevalence. Thus, this analysis of currently available domestic data can provide information to the current screening practice before mortality outcome studies are available in China (which will not be very soon). The failure of small trials in Europe to detect any reduction in mortality has usually been attributed to insufficient in subject number (fewer than 2000 in low-dose CT arms) [7–9]. Only the statistically powered NLST study, with a sample size of 53,454, has detected a 20% mortality reduction with low-dose CT screening over chest X-ray screening [5], which was why we simulated 100,000 participants to obtain robust results. The relatively smaller reduction in lung cancer deaths of 17.2% with low-dose CT screening over chest X-ray screening in the present study compared with the 20% in the NLST may be attributable to differences in enrollment criteria [40] (such as smokers in the present study and heavy smokers in the NLST), other population characteristics, and quality of healthcare services.

In the present study, sensitivity analysis showed that the prevalence of lung cancer in the screened cohort was the factor with the strongest influence on reduction in lung cancer deaths, indicating that the selection criteria for eligibility for lung cancer screening programs should be carefully and rigorously defined [40]. Although higher upper age limits for screening eligibility were related with greater reduction in mortality, individuals aged 75 years and older are more vulnerable than younger persons to clinical interventions such as invasive diagnostic and treatment procedures and are at high risk of death from other diseases (such as cardiac arrest and stroke). Thus, screening among this old population potentially affects the actual benefit from screening and may lead to ethical issues. Conversely, enrolling young individuals in screening programs results in only small gains from the perspective of screening service providers. Thus, a balance between risk and gain should be sought when selecting the age range to be screened. We recommend a mid-range spread, say 55–75 years: this age range has a relatively high screening benefit (24.5% reduction in lung cancer deaths with low-dose CT screening over no screening) and provides a relatively high coverage of those likely to have lung cancer (63.9% of those aged 45–80 years). As to the smoking criteria, we found that reductions in lung cancer deaths increase in parallel with the proportion of smokers in both men and women in the smoking cohort. We therefore strongly recommend consideration of low-dose CT screening for lung cancer in regions with high tobacco smoking rates, whereas regions with limited investment in screening should primarily target smokers. Additionally, the reduction in lung cancer deaths was not quite pronounced in this study with the relaxed inclusion criteria. It is noteworthy that when the emphasis is on heavy smokers and other criteria for screening are imposed (such as those in the Chinese Consensus on Early Diagnosis of Primary Lung Cancer [20]), a greater screening benefit can be expected compared with nonselective criteria [40]. Considering the important contribution of lung cancer prevalence to screening benefit, a combination of other methods such as biomarkers to identify high-risk individuals would also likely to increase screening benefits [41].

Regarding other factors that influenced screening effects, the sensitivity of low-dose CT had a relatively important positive influence on the reduction in lung cancer deaths; so was the proportion of early-stage cancer among lung cancers detected with low-dose CT screening, reinforcing the need to develop highly sensitive screening techniques [42]. The positive relationship between survival possibility after early treatment of cancer and reduction in deaths emphasizes the need to improve the effectiveness of early treatment to achieve better prognoses and screening benefits. Given the effect of acceptance of treatment on outcomes, screening programs should also consider incorporating approaches to enhancing willingness and financial ability of patients with lung cancer to undergo recommended treatment, particularly in developing countries. Additionally, our findings indicate that survival rates of non-early-stage cancer patients have a very limited impact on mortality outcomes.

For individuals who do not have lung cancers, false positive results remain the most critical issue of low-dose CT screening [43–45]. This is reinforced by the higher numbers of false diagnoses and deaths due to false diagnosis with low-dose CT screening compared with chest X-ray screening in the present study. Because the specificity of low-dose CT directly influences such outcomes, further efforts should be put into increasing the discriminative ability of imaging techniques and their computer-aided diagnostic systems [46], developing optimal diagnostic thresholds [44, 45], and employing other imaging techniques (such as positron emission tomography) [47] or other non-imaging approaches (such as highly specific biomarkers) [41, 48, 49]. Additionally, standardization of workflow for diagnosis and treatment, in addition to screening, is indispensable to eliminating unnecessary cost and morbidities associated with lung cancer screening programs [50].

In our study, gains in QALYs of low-dose CT screening over chest X-ray screening and no screening were less impressive than reduction in lung cancer deaths. This was mainly because unscreened individuals with lung cancer can enjoy a higher quality of life during the symptom-free interval before diagnosis of their cancers, whereas individuals with false-positive diagnoses experience a decreased quality of life as a result of anxiety and unnecessary diagnosis or treatment. For example, in the base-case analysis, these two sources resulted in loss of 562 QALYs in the low-dose CT screening scenario than in the no screening scenario (counterbalanced primarily by the stage-shift benefit of 812 QALYs, which led to the final 250-year gain). Thus, close attention should also be paid to physiological reactions if we wish to provide participants in the low-dose CT screening scenario with better quality of life [51].

Limitations remain in this study. First, the decision tree we used is a simplified representation of complex clinical paths and individuals’ behaviors. For example, we dichotomized the distribution of cancer stages into early and non-early; full I–IV staging (especially with associated differences in survival) would have made the model more accurate. This was mainly because of lack of large domestic studies from which we could draw robust estimates of screening and clinical parameters. Additionally, the absence of transition possibilities in lung cancer stage among the extremely heterogeneous histological types of lung cancer prevented us from employing stage transition Markov models. Second, for the same reasons, we only considered baseline screening in this study, whereas some programs in China already adopted a baseline and annual repeat screening fashion [52], in which higher proportion of early-stage lung cancers in repeat rounds could be expected. Thus, longitudinal screening parameters from such programs and their impact on mortality reduction are highly needed for further decision analyses. Third, although false diagnoses, missed diagnoses, and over-diagnoses of indolent cancers were considered, we did not consider complex issues such as passive smoking and competing risk from other diseases in old individuals, but left these for future studies. Finally, the lack of expenditure data at the national level and differences in costs of lung cancer treatment between different regions thus far prevents evaluation of the cost-effectiveness of lung cancer screening. Thus, the balance between input and yield requires further in-depth evaluation.

## Conclusions

Mortality outcome derived from this decision analysis were in favor of conducting low-dose CT screening in urban China for early detection and treatment of lung cancer. However, for nationwide promotion in the future, further work should be done to optimize the effects of screening, minimize false positive diagnoses, and improve participants’ actual quality of life, such as by developing selection criteria for screening that are more appropriate to Chinese participants, standardizing diagnostic and treatment methods, and adapting screening protocols according to availability of funding and other local conditions.
